# The regulatory mechanism of LncRNA-mediated ceRNA network in osteosarcoma

**DOI:** 10.1038/s41598-022-11371-w

**Published:** 2022-05-24

**Authors:** Chengsen Lin, Jifeng Miao, Juliang He, Wenyu Feng, Xianxiang Chen, Xiaohong Jiang, Jianhong Liu, Boxiang Li, Qian Huang, Shijie Liao, Yun Liu

**Affiliations:** 1 Department of Orthopedics, The First Affiliated Hospital of Guangxi Medical University, Nanning, China; 2 Department of Orthopedics, The Children’s Hospital of Guangxi Zhuang Autonomous Region, Maternal and Child Health Hospital of Guangxi Zhuang Autonomous Region, Nanning, China; 3 Department of Bone and Soft Tissue Neurosurgery, Affiliated Tumor Hospital of Guangxi Medical University, Nanning, China; 4 Department of Orthopedics, Ethnic Hospital of Guangxi Zhuang Autonomous Region, Nanning, China

**Keywords:** Bone cancer, Cancer genomics, RNA sequencing

## Abstract

Aberrantly expressed lncRNAs have been reported to be closely related to the oncogenesis and development of osteosarcoma. However, the role of a dysregulated lncRNA-miRNA-mRNA network in osteosarcoma in the same individual needs to be further investigated. Whole transcriptome sequencing was performed on the tumour tissues and matched paratumour tissues of three patients with confirmed osteosarcoma. Two divergent lncRNA-miRNA-mRNA regulatory networks were constructed in accordance with their biological significance. The GO and KEGG analysis results of the mRNAs in the two networks revealed that the aberrantly expressed lncRNAs were involved in regulating bone growth and development, epithelial cell proliferation, cell cycle arrest and the N-terminal acetylation of proteins. The survival analysis results of the two networks showed that patients with high expression of *GALNT3*, *FAM91A1*, *STC2* and *SLC7A1* end in poorer prognosis. Likewise, patients with low expression of *IGF2*, *BLCAP*, *ZBTB47*, *THRB*, *PKIA* and *MITF* also had poor prognosis. A subnetwork was then constructed to demonstrate the key genes regulated by aberrantly expressed lncRNAs at the posttranscriptional level via the ceRNA network. Aberrantly expressed lncRNAs in osteosarcoma tissues regulate genes involved in cellular proliferation, differentiation, angiogenesis and the cell cycle via the ceRNA network.

## Introduction

Osteosarcoma, the most common malignant primary bone tumour in adolescents, is characterized by immature osteoid formation, abnormal osteoblastic differentiation, early lung metastasis, high recurrence, and a high mortality rate^1^. The application of neoadjuvant chemotherapy has improved the prognosis of patients with osteosarcoma and has therefore become a widely recognized standard of osteosarcoma treatment. However, recent studies have reported a bottleneck in the clinical application of these regimens, as the 5-year survival rate of patients with osteosarcoma has not improved in the past 20–30 years and has hovered at approximately 55–75%^2^. Tumorigenesis is often accompanied by abnormal gene expression, which has provided a novel therapeutic strategy for targeting abnormally expressed genes in various malignant tumours, such as lung cancer^3^, breast cancer^4^, gastric cancer^5^, and kidney cancer^6^, and produced remarkable results. Although many studies have explored targeted therapies for osteosarcoma, most are based on clinical experience and have not provided clear and effective therapeutic targets or ideal therapeutic drugs that can remarkably improve prognosis^7^. Therefore, further studies on the regulatory mechanism of osteosarcoma-related genes are needed to provide a basis for the development of novel therapeutic plans.

Noncoding RNAs (ncRNAs) play important roles in the regulation of gene expression at multiple levels and are thus involved in the occurrence and progression of malignant tumours^8^. Among them, the proposal of the ceRNA theory in 2011 illustrated the precise regulation of oncogenes and tumour suppressor genes by various ncRNAs from the entire transcriptome at the posttranscriptional level. In this system, miRNAs bind to mRNAs through compensatory sequences, called miRNA response elements (MREs), to prohibit them from translating into proteins, which results in the suppression of the target gene. NcRNAs, such as long noncoding RNAs (lncRNAs), circRNAs, and pseudogenes, can competitively bind to miRNAs via shared MREs. As a result, the miRNAs blocked by these ncRNAs were not able to inhibit mRNAs since their MRE binding sites were occupied. In total, these ncRNAs could weaken the inhibitory effect of miRNAs on targeted genes and eventually recover target gene function. These ncRNAs were defined as endogenous competitive RNAs (ceRNAs)^9,10^. As a result, these ncRNAs, miRNAs, and mRNAs constitute the ceRNA–miRNA–mRNA network. The ceRNA–miRNA–mRNA network is a large-scale and complex posttranscriptional regulatory network because many miRNAs share the same MREs with multitude ncRNAs, as well as with multitude mRNA^10^. LncRNA, an important kind of ceRNA, regulates gene expression at multiple levels and is also involved in the regulation of various biological characteristics, such as cellular proliferation, apoptosis, and tumour metastasis^11^. Recently, they have attracted much interest, especially in predicting their potential functions by their unique sequence^12^. Studies have reported that lncRNAs exhibit evidently abnormal expression in various kinds of tumours, leading to an abnormal ratio of miRNAs/lncRNAs affecting the protein coding process of mRNAs. If these mRNAs are involved in tumorigenesis, tumours may occur^12,13^. Therefore, it is extremely crucial to unveil the aberrant lncRNA-miRNA-mRNA network in osteosarcoma.

Previous studies have confirmed that multiple lncRNAs as ceRNAs can regulate the biological characteristics of osteosarcoma cells. For example, LINC00588 can inhibit the migration, invasion, endothelial cell function, and epithelial–mesenchymal transition (EMT) of osteosarcoma cells through the LINC00588/miR-1072/TP53 axis^14^. GAS5 also inhibits the proliferation and migration of osteosarcoma cells via the GAS5/miR-663a/MYL9 axis^15^. In contrast, LINC00839 promotes the proliferation, migration, and invasion of osteosarcoma cells via the LINC00839/miR-454-3p/c-met axis^16^. LINC01128 can also promote the proliferation, migration, and invasion of osteosarcoma cells via the LINC01128/miR-299-3p/MMP2 axis^17^. UCA1 mediates EMT and activates the PI3K/Akt/mTOR pathway via the UCA1/miR-582/CREB1 axis to promote osteosarcoma metastasis^18^. In addition, MEG3 participates in the development of drug resistance in osteosarcoma through the MEG3/hsa-miR-200b-3p/AKT2 axis^19^. Moreover, several dysregulated lncRNAs could affect multiple key signalling pathways in osteosarcoma cells that are closely related to osteosarcoma recurrence^20^. These reports suggest that aberrantly expressed lncRNAs play important roles in the development of osteosarcomas through a ceRNA mechanism.

However, from these results, it is difficult to systematically explain the dysregulation of the lncRNA-miRNA-mRNA network in osteosarcoma because most of the studies integrated lncRNA, miRNA, and mRNA expression profiles from different tumour specimens or only performed RNA sequencing on osteosarcoma cell lines. In addition, the majority of ceRNA studies on osteosarcoma are limited to individual ceRNA axes. A ceRNA network is large-scale and complex; therefore, the inhibitory effects on osteosarcoma cell lines are difficult to realize from a single ceRNA axis. In-depth RNA sequencing conducted by Lin Xie et al. by building a lncRNA-miRNA-mRNA network using primary lesions, lung metastases, and normal tissue samples from a patient with osteosarcoma revealed the genes and pathways responsible for the occurrence and metastasis of osteosarcoma^21^. However, the samples they obtained from different individuals resulted in some degree of individual variation. Thus, the systemic regulatory effects of the lncRNA-miRNA-mRNA network on osteosarcoma-related genes need further exploration.

Thus, we performed whole transcriptome sequencing on the tumour tissues and matched paratumour tissues of three patients with osteosarcoma and then extracted differentially expressed lncRNAs, miRNAs, and mRNAs to construct lncRNA-miRNA-mRNA networks to reveal the regulation of abnormally regulated lncRNAs on osteosarcoma-related genes. GO and KEGG analyses of the mRNAs in the network were also performed to reveal the aberrantly expressed lncRNAs that regulate the functions of osteosarcoma-related genes. Kaplan–Meier survival analysis of mRNAs in the network was also performed using the survival data of 85 patients with confirmed osteosarcoma from the TARGET database. The key osteosarcoma-related genes that could potentially be regulated by aberrantly expressed lncRNAs and have a potential impact on survival were screened and then used to construct a ceRNA subnetwork to investigate the regulatory mechanism of abnormal lncRNAs as ceRNAs. The flowchart of the whole procedure is shown in supplementary Fig. 1.

## Methods

### Sample preparation

This study was conducted in accordance with the 1964 revised Helsinki Declaration and was approved by the Ethics Committee of The First Affiliated Hospital of Guangxi Medical University (approval no. 2019KY-E-097). Written informed consent was obtained from the patients prior to tissue acquisition. Tumour and matched paratumour tissue samples were obtained from three patients with confirmed osteosarcoma who underwent surgery at the First Affiliated Hospital of Guangxi Medical University (Nanning, P. R China). RNAfollow^@^ Tissue Stabilization Solution (NCM Biotech, China) was immediately added to the acquired tissue at a temperature of 4 °C overnight before being transferred for storage at − 80 °C.

For RNA extraction, 30 mg tissue was added to liquid nitrogen and then ground using OMEGA E.Z.N. A Total RNA Kit I (OMEGA BIO-TEK, USA). After RNA extraction, nondenaturing agarose gel electrophoresis and spectrophotometry (NanoDrop™ One/One^C^, Thermo Fisher Scientific, Inc., USA) were used to measure the total amount of RNA, and RNA integrity was assessed using an Agilent 2100 bioanalyser (Agilent Technologies, Inc., Germany). Follow-up tests were carried out when the sample quality met the requirements of the sequencing quality for database construction.

### LncRNA and mRNA library construction, sequencing, and raw data processing

Ribosomal RNA was removed from the total RNA, and the remaining RNA was broken into short fragments at random. First-strand cDNA was synthesized using random hexamers with six base pairs and RNA fragments as templates. Second-strand cDNA was synthesized by adding buffer solution, dNTPs, RNase H, and DNA polymerase I. Then, the second-strand cDNA was purified with a QIAquick polymerase chain reaction (PCR) purification kit (QIAGEN, Germany), eluted with EB buffer solution, and repaired with terminal, base A, and sequencing joints. The second cDNA chain was then degraded using uracil-N-glycosylase (R&D Systems, USA). Then, fragment size was selected by agarose gel electrophoresis, and PCR amplification and cDNA library construction were finished. Finally, the library was sequenced on an Illumina X Ten/Nova™ (Illumina, Inc., USA) platform according to the manufacturer’s instructions to obtain the raw data.

The raw data were filtered to obtain clean data with ActivePerl (Version 5.24.1, Perl Foundation) scripts. The transcriptome data-matching software TopHat2 was then used to compare the filtered ribosome reads to the reference genome to obtain an alignment file of BAM format. The Cufflinks reference annotation-based transcripts were then used to assemble the transcripts. We measured the number of exons, length, annotation and expression from the assembled transcripts to obtain candidate lncRNAs and their characteristics. The identified sequences of lncRNAs, mRNAs, and transcripts of unknown coding potential were then quantified, and output the counts data matrix in ENST ID.

### Small RNA library construction, sequencing, and raw data proceeding

A 3 μg sample from the Small RNA Sample Pre-Kit (Illumina, Inc., USA) was employed to construct a library in accordance with the manufacturer’s instructions. Connectors were then added to the 5′ and 3′ ends of small RNAs to synthesize cDNA via reverse transcription. Afterwards, PCR amplification, separation of the target DNA fragments using polyacrylamide gel electrophoresis, and the construction of a small RNA cDNA library via gel cutting and recovery were performed.

Qubit2.0 was employed for preliminary quantification, and the library was diluted to 1 ng/μl. The INSERT size of the library was then spotted using an Agilent 2100. Once the INSERT size met the expectations, qPCR analysis was employed to quantify the effective concentrations of the library accurately to meet the requirements of the library machine. After the library qualified our inspections, clustering of the index-coded samples was processed using the TruSeq SR Cluster Kit v3-cBot-HS (Illumina, Inc., USA) of the cBot Cluster Generation System (Illumina, Inc., USA) in accordance with the instructions of the manufacturer. After the clusters were generated, the library preparations were sequenced using the Illumina HiSeq 2500™ platform (Illumina, Inc., USA), and two 50 bp single-end reads were generated.

Raw fastq format data were then performed using made-to-order ActivePerl and Python (Version 3.8, Python Software Foundation) scripts for clean reads. Small RNA tags were then mapped to reference sequences as described by Bowtie^22^. The mapped small RNA tags were employed for searching and identifying known miRNAs with the reference from miRBase20.0. The modified software miRDeep2 was used to quantify the known miRNA counts^23^. miREvo^24^ and miRDeep2 were then integrated to predict potential novel miRNAs.

### Differential expression analysis

Use perl script to convert ENST ID to gene name based on Ensembl database (version GRCh38.89, updated 2020-03), and then separate lncRNA and mRNA data. Combine data with the same gene name. The EdgeR package of the R programming language was then used for normalization and differential expression analysis according to the edgeR user guide. Genes with a fold change > 2 and an adjusted false discovery rate (FDR) using the Benjamini‑Hochberg method of p < 0.05 were defined as differentially expressed genes (DEGs). All types of transcripts (lncRNAs, miRNAs, mRNAs) were used for the overall differential analysis, and the differentially expressed transcripts were plotted in a volcano plot with log2(fold change) as the abscisic coordinate and − log10(FDR) as the ordinate. The expression heatmaps of all differential transcripts were drawn using the gplots package. The abscissa is the sample name; the ordinate is the gene symbol; the expression is expressed by log10 (normalizedData + 0.001), and the colour key and histogram are attached.

### Construction of the ceRNA regulatory network and functional analysis

LncRNA–miRNA regulatory pairs were selected after the screening of DElncRNAs from the associations in ENCORI (http://starbase.sysu.edu.cn/). Additionally, miRNA–mRNA regulatory relationships were investigated based on the regulatory relation in TargetScan (Release 7.2, http://www.targetscan.org/vert_72/), miRTarBase (http://mirtarbase.cuhk.edu.cn/), and miRDB (http://mirdb.org/). Then, Cytoscape (version 3.8.2, Cytoscape Consortium) was used to integrate the lncRNA-miRNA regulatory pairs and miRNA–mRNA regulatory pairs and finally construct the lncRNA-miRNA–mRNA network. The genes in the constructed ceRNA regulatory network were then used for further GO and KEGG analyses using clusterprofile in R.

### Survival analysis

The expression profile data of mRNAs in osteosarcoma tissues were compared with the clinical data from the downloaded GDC TARGET-OS database (https://gdc.xenahubs.net) to screen the key mRNAs regulated by the ceRNA network. Kaplan–Meier curves in R software were used for overall survival analysis. Statistical analysis was processed using the log-rank test. The threshold for survival prognosis significance was confirmed if p < 0.05. The final subnetworks were constructed based on these mRNAs.

## Results

### Clinical data

**Figure 1:**
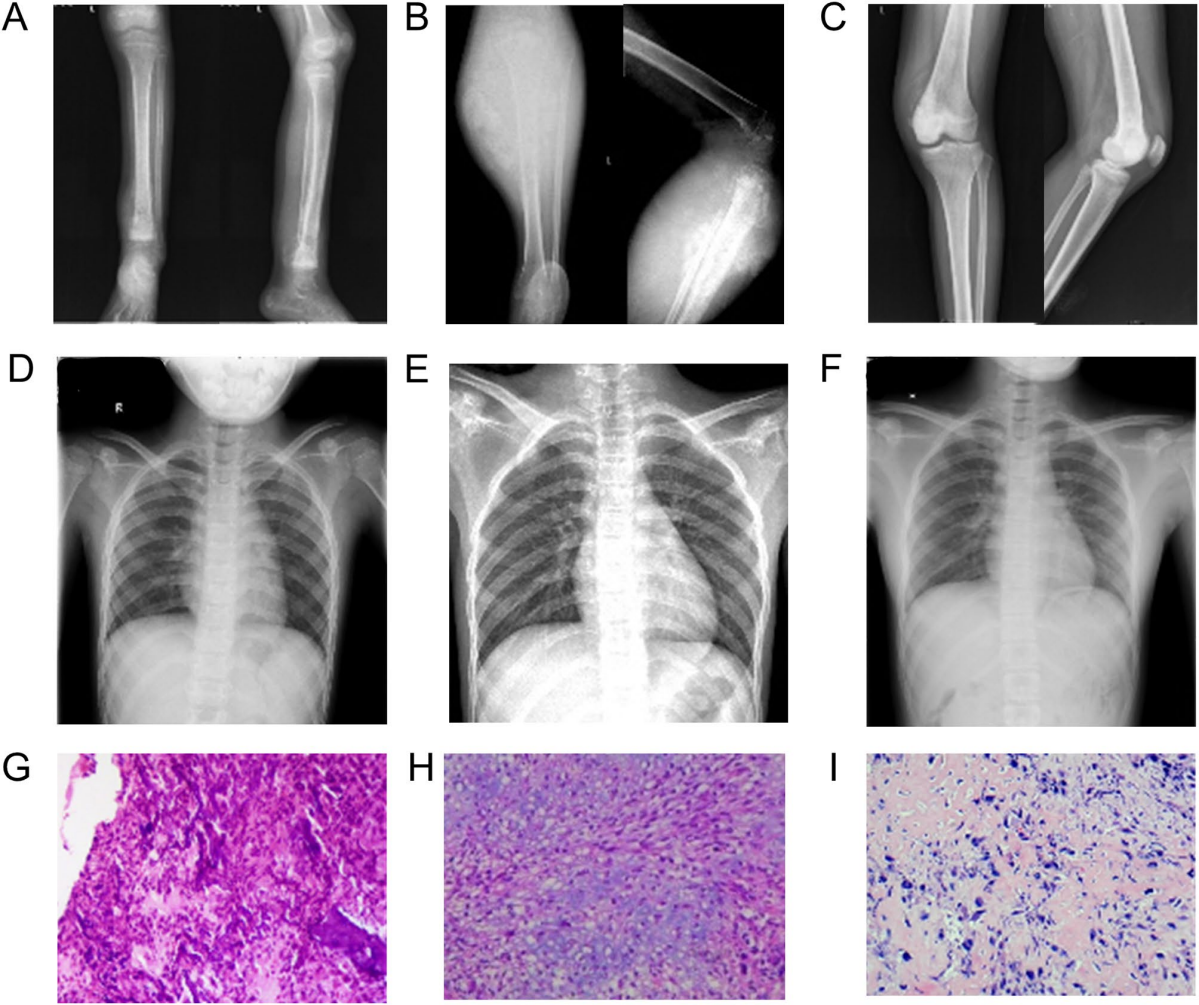
X Ray and the pathological result. X Ray of tibia (**A**) and chest (**D**) and the pathology (**G**) of P1. X Ray of proximate tibia (**B**) and chest (**E**) and the pathology (**H**) of P2. X Ray of knee (**C**) and chest (**F**) and the pathology (**I**) of P3.

The clinical information of three patients with osteosarcoma is listed in Table 1. All of them were diagnosed by pathological analysis. The patients were 7–16 years old. Tumour and paratumour tissues were obtained for RNA-seq when conducting the biopsy. X-rays of the primary tumour and chest and pathological images are shown in Fig. 1.

**Table 1:** Patient baseline data.

	P1	P2	P3
Gender	Female	Female	Female
Age (years old)	7	12	16
Enneking stage	IIb	IIb	IIb
Metastasis	No	No	No

### Screening of differentially expressed mRNAs, miRNAs, and lncRNAs

**Figure 2:**
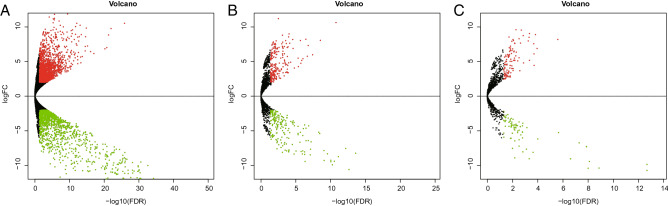
Volcano Plot of DEmRNAs, DElncRNAs and DEmiRNAs. (**A**) DEmRNAs. (**B**) DElncRNAs. (**C**) DEmiRNAs. Upregulated genes are marked in light red; downregulated genes are marked in light green. (DEmRNAs, DElncRNAs and DEmiRNAs were selected with thresholds of fold change > 2 and p < 0.05).

**Figure 3:**
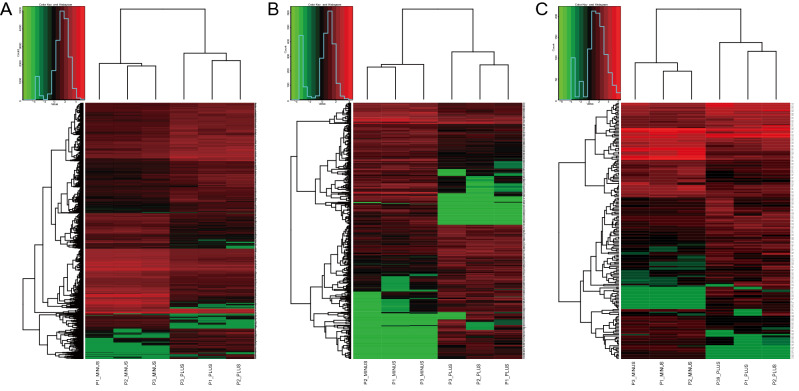
Heatmaps of DEmRNAs, DElncRNAs and DEmiRNAs. (**A**) DEmRNAs. (**B**) DElncRNAs. (**C**) DEmiRNAs. Upregulated genes are marked in light red; downregulated genes are marked in light green. (DEmRNAs, DElncRNAs and DEmiRNAs were selected with thresholds of fold change > 2 and p < 0.05).

We sequenced the whole transcriptomes of three osteosarcoma and three paratumour tissues by RNA-seq and identified 79,768 mRNAs, 3834 lncRNAs, and 1960 miRNAs. Subsequently, 414 DElncRNAs, 184 DEmiRNAs, and 3275 DEmRNAs were screened under fold change > 2 and p < 0.05. Among them, 220 lncRNAs, 104 miRNAs, and 1849 mRNAs were upregulated, while 194 lncRNAs, 80 miRNAs, and 1426 mRNAs were downregulated (Figs. 2 and 3). Supplementary Tables S1, S2, and S3 list the 20 lncRNAs, miRNAs, and mRNAs with the largest expression differences, respectively (up- and down-regulation).

### LncRNA–miRNA–mRNA ceRNA network

**Figure 4:**
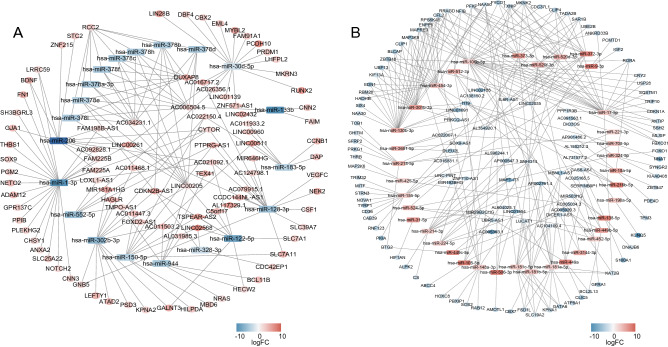
The lncRNA-miRNA-mRNA competing endogenous RNA network. (**A**) ceRNA network with high-low–high trend. (**B**) ceRNA network with low–high-low trend. Rhombus indicates lncRNAs, rectangle indicates miRNAs, and oval indicates mRNAs. Orange-red indicates high expression, and blue indicates low expression. The darker the color, the higher or lower expression.

Sixty-three target DEmiRNAs of DElncRNAs were predicted by ENCORI. Next, 1703 target mRNAs of these 63 DEmiRNAs were predicted in TargetScan, miRDB, and miRTarBase. A total of 275 target DEmRNAs were obtained for network construction by using the intersection of the predicted target mRNAs with DEmRNAs. We divided the network into high–low–high and low–high–low networks according to the direction of differential expression to obtain the network with biological significance. There were 114 nodes, 169 edges and 15 hubs (interactions > 5) in the high–low–high network, including 40 lncRNAs, 20 miRNAs, and 54 mRNAs (Fig. 4A), and 163 nodes and 310 edges and 24 hubs (interactions > 5) in the low–high–low network, including 40 lncRNAs, 37 miRNAs, and 86 mRNAs (Fig. 4B).

### Function of the mRNAs regulated by the ceRNA network

**Figure 5:**
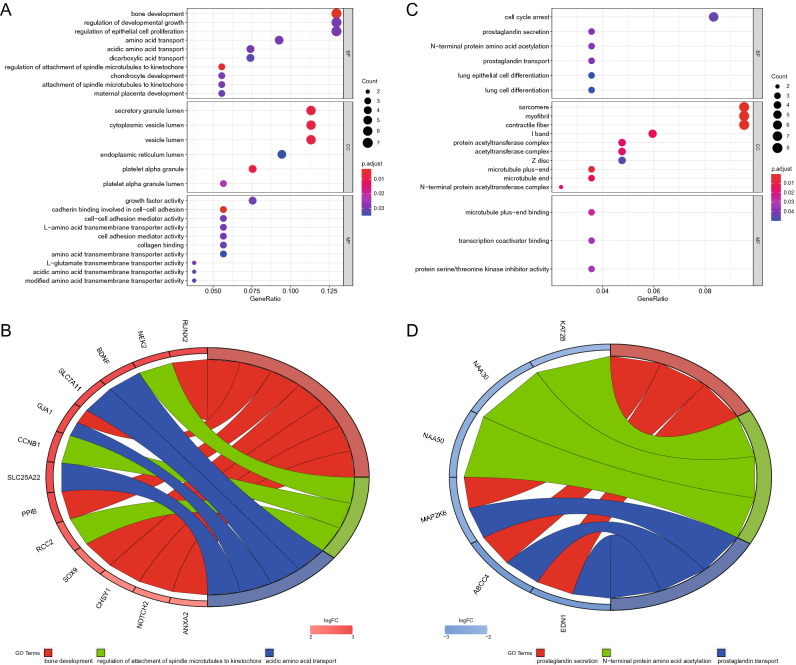
GO enrichment analysis of DEmRNAs in the ceRNA network. The GO enrichment bubble plot (**A**) and GO chord plot (**B**) of DEmRNAs in the high-low-high trending ceRNA network. The GO enrichment bubble plot (**C**) and GO chord plot (**D**) of DEmRNAs in the low–high-low trending ceRNA network (BP: biological processes. CC: cellular components. MF: molecular functions. A P < 0.05 was considered to indicate a statistically significant difference).

**Figure 6:**
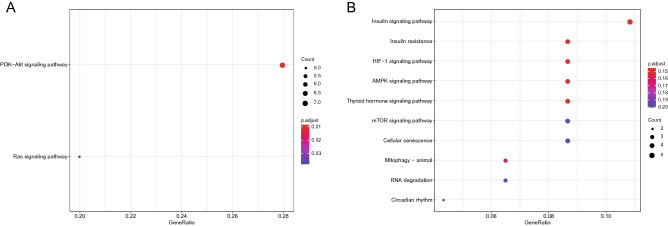
KEGG enrichment analysis of DEmRNAs in the ceRNA network. (**A**) The KEGG enrichment bubble plot of DEmRNAs in the high-low-high trending ceRNA network. (**B**) The KEGG enrichment bubble plot of DEmRNAs in the low–high-low trending ceRNA network (A P < 0.05 was considered to indicate a statistically significant difference).

We performed Gene Ontology (GO) and Kyoto Encyclopedia of Genes and Genomes (KEGG) enrichment of the mRNAs in the network to elucidate the role of lncRNA–miRNA–mRNA in the development of osteosarcoma. The 54 genes in the high–low–high network were involved in ten biological processes (BPs), namely, “bone development”, “regulation of developmental growth”, “regulation of epithelial cell proliferation”, “amino acid transport”, “acidic amino acid transport”, “dicarboxylic acid transport”, “regulation of attachment of spindle microtubules to kinetochore”, “chondrocyte development”, “attachment of spindle microtubules to kinetochore”, and “maternal placenta development”. Six cellular components (CCs) included “secretory granule lumen”, “cytoplasmic vesicle lumen”, “vesicle lumen endoplasmic reticulum lumen”, “platelet alpha granule”, and “platelet alpha granule lumen”. Ten molecular functions (MFs) were “growth factor activity”, “cadherin binding involved in cell–cell adhesion”, “cell–cell adhesion mediator activity”, “l-amino acid transmembrane transporter activity”, “cell adhesion mediator activity”, “collagen binding”, “amino acid transmembrane transporter activity”, “l-glutamate transmembrane transporter activity”, “acidic amino acid transmembrane transporter activity”, and “modified amino acid transmembrane transporter activity” (p < 0.05, Fig. 5A,B). KEGG analysis revealed that these genes were associated with two pathways, namely, the “P13K-Akt signalling pathway” and “Ras signalling pathway” (p < 0.05, Fig. 6A). In addition, the 86 genes in the low–high–low network were involved in six BPs, namely, “cell cycle arrest”, “prostaglandin secretion”, “N-terminal protein amino acid acetylation”, “prostaglandin transport”, “lung epithelial cell differentiation”, and “lung cell differentiation”. Six CCs included “sarcomere”, “myofibril”, “contractile fibre”, “protein acetyltransferase complex”, “acetyltransferase complex”, “microtubule plus-end”, “microtubule end”, and “N-terminal protein acetyltransferase complex”. Three MFs were “microtubule plus-end binding”, “transcription coactivator binding”, and “protein serine/threonine kinase inhibitor activity” (p < 0.05, Fig. 5C,D). However, these genes were not substantially enriched in any KEGG pathway (p > 0.05, Fig. 6B).

### Survival analysis and subnetwork of survival-associated mRNAs

**Figure 7:**
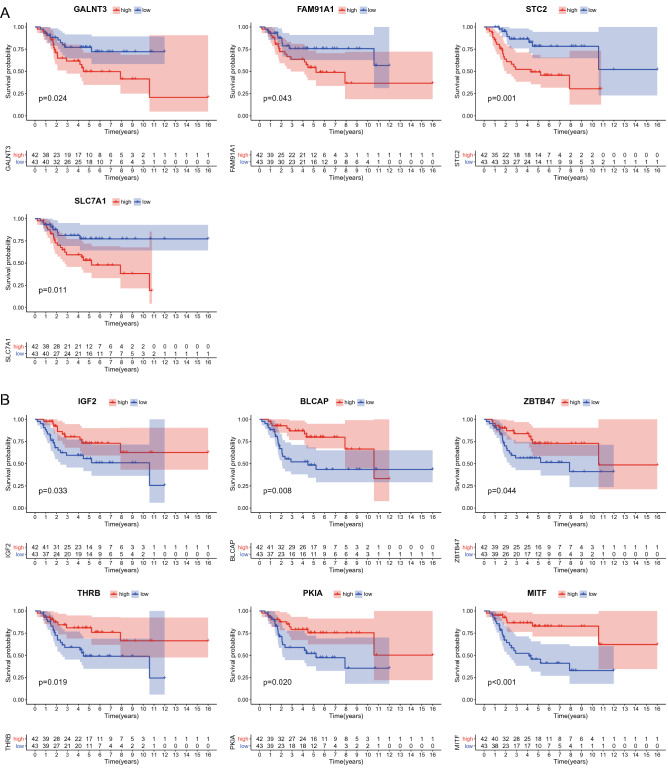
Kaplan–Meier survival analysis of the DEmRNAs in the ceRNA network. The TARGET database was used to analyse the survival prognosis of 85 mRNAs in osteosarcoma patients. (**A**) Increased expression levels of GALNT3, FAM91A1, STC2 and SLC7A1 were associated with poor prognosis. (**B**) Lower expression levels of IGF2, BLCAP, ZBTB47, THRB, PKIA and MITF were associated with poor prognosis (A P < 0.05 was considered to indicate a statistically significant difference).

**Figure 8:**
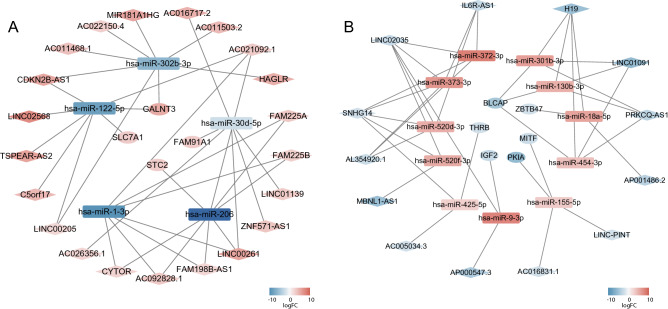
Construction of the lncRNA-miRNA-mRNA subnetwork based on mRNAs with survival prognostic potential. (**A**) ceRNA network with high-low–high trend. (**B**) ceRNA network with low–high-low trend. Rhombus indicates lncRNAs, rectangle indicates miRNAs, and oval indicates mRNAs. Orange-red indicates high expression, and blue indicates low expression. The darker the color, the higher or lower expression.

We performed Kaplan–Meier survival analysis on the mRNAs in the network using the data of 85 patients with osteosarcoma from the TARGET database to obtain the key genes and the corresponding lncRNA–miRNA–mRNA subnetworks. We found that the high expression of *GALNT3, FAM91A1, STC2,* and *SLC7A1* (Fig. 7A) and the low expression of *IGF2, BLCAP, ZBTB47, THRB, PKIA,* and *MITF* (Fig. 7B) may result in the poor prognosis of patients. For further study, we constructed a subnetwork using the survival-associated mRNAs (Fig. 8A and B).

## Discussion

Osteosarcoma is a highly malignant tumour of the bone. However, the prognosis of patients with advanced osteosarcoma has not shown recent improvements. Therefore, searching for the regulatory mechanisms of osteosarcoma cells to unveil and provide a theoretical basis for the development of novel therapeutic strategies that could improve the prognosis of patients is important^2,25^. MiRNAs are known to silence gene expression, while lncRNAs, which can be used as miRNA sponges, can reverse the inhibitory effects of miRNAs on mRNAs and have a great influence on tumour progression and biological features^26,27^. However, most of the previous studies of lncRNA-miRNA-mRNA regulatory mechanisms in osteosarcoma were based on lncRNAs, which play a key role in other malignant tumours. These studies further confirmed the abnormal expression of lncRNAs in osteosarcoma tissues and verified their effect on the level of osteosarcoma cells and animal models^28^. To systematically uncover the lncRNA-miRNA-mRNA regulatory mechanism, we conducted high-throughput sequencing of lncRNAs, mRNAs, and miRNAs in three pairs of osteosarcoma and matched paratumour tissues and constructed ceRNA networks to identify the key genes regulated by lncRNAs and systematically elaborate the regulation of osteosarcoma-related genes by the lncRNA-miRNA-mRNA network. A total of 414 differential lncRNAs, 184 differential miRNAs, and 3275 differential mRNAs were screened. According to the biological features of ceRNAs, we constructed two ceRNA networks for the differentially expressed RNAs with different abnormal expression trends. The high–low–high trend network included 37 lncRNAs, 20 miRNAs, and 54 mRNAs, and the low–high–low trend network included 40 lncRNAs, 20 miRNAs, and 54 mRNAs.

LncRNAs exert regulatory effects by affecting mRNAs encoding proteins; hence, survival-related mRNAs are crucial in the ceRNA network. We performed GO and KEGG analyses using the mRNAs in the network to clarify the functions played by lncRNAs in the ceRNA network. GO analysis indicated that the upregulated genes in the network were involved in the regulation of bone development, growth, and epithelial cell proliferation. The pathological features of abnormal osteogenic differentiation in osteosarcomas support the results. Genes involved in epithelial cell proliferation, such as *BCL11B*, *NRAS*, *THBS1*, and *VEGFC*, promote angiogenesis and tumorigenesis, reflecting the heterogeneity of osteosarcoma-related genes. In comparison, the downregulated genes in the network were involved in cell cycle arrest and N-terminal amino acid acetylation. The inhibition of genes involved in cell cycle arrest corresponds to the abnormally increased proliferation of osteosarcoma cells. In addition, N-terminal acetylation has a catalytic role in cancers^29^. However, no studies have reported on its mechanism. The results of our KEGG analysis revealed that the main upregulated genes in the network were involved in the PI3K–Akt and RAS signalling pathways, both of which are involved in the growth and metastasis of osteosarcoma^30–32^. Thus, our results suggest that the lncRNAs in the network play an important role in regulating the occurrence and progression of osteosarcomas.

We performed a survival analysis using the mRNA expression profiles and survival data of 85 confirmed cases of osteosarcoma from the TARGET database to screen the key genes regulated by lncRNAs. We found that patients with high expression of *GALNT3, FAM91A1, STC2,* and *SLC7A1* had poor prognosis, whereas patients with low expression of *IGF2, BLCAP, ZBTB47, THRB, PKIA,* and *MITF* had poor prognosis. Subnetworks were therefore constructed based on these key genes to demonstrate the regulatory mechanism of the aberrantly expressed lncRNAs.

Twenty-one lncRNAs (CDKN2B-AS1, AC011468.1, AC022150.4, MIR181A1HG, AC16717.2, AC011503.2, AC021092.1, HAGLR, FAM225A, FAM225B, LINC01139, ZNF571-AS1, LINC00261, FAM198B-AS1, AC092828.1, CYTOR, AC026356.1, CO5ORF71, LINC00205, TSPEAR-AS2, LINC02568) and 5 miRNAs (hsa-miR-122-5p, hsa-miR-320b-3p, hsa-miR-206, hsa-miR-1-3p, and hsa-miR-30d-5p) comprised the high–low–high subnetwork. The mRNAs regulated by the ceRNA network included *SLC7A1, GALNT3, FAM91A1,* and *STC2. SLC7A1* mediates the uptake of arginine by cancer cells and improves the survival of cancer cells by inhibiting apoptosis^33,34^. *GALNT3* can glycosylate *MUC1*, which further activates the PI3K/Akt pathway and promotes tumour proliferation and invasion^35^. *STC2*, a member of the glycoprotein hormone-secreting family, promotes the differentiation and mineralization of osteoblasts^36,37^; inhibits apoptosis; promotes resistance to oxidative damage; promotes the proliferation, survival, and migration of tumour cells; and thus promotes tumour progression^38^. Whether *FAM91A1* is related to tumours has not yet been reported. However, we found that *FAM91A1* is a risk factor for osteosarcoma; thus, its functions need to be further studied. Above all, the aberrantly upregulated lncRNAs regulate the genes involved in key biological characteristics, such as apoptosis inhibition, survival, migration, invasion, and proliferation of tumour cells.

The low–high–low subnetwork consisted of 13 lncRNAs (SNHG14, LINC02035, IL6R-AS1, PRKCQ-AS1, H19, LINC01091, MBNL1-AS1, AP000547.3, AL354920.1, AC016831.1, LINC-PINT, AC005034.3, and AP001486.2) and 12 miRNAs (hsa-miR-9-3p, has-miR-18a-5p, has-miR-372-3p, has-miR-373-3p, has-miR-301b-3p, has-miR-520d-3p, has-miR-520f-3p, has-miR-520f-3p, has-miR-520f-3p, has-miR-155-5p, has-miR-454-3p, and has-miR-425-5p). The mRNAs regulated by these lncRNAs via the ceRNA network include *MITF, THRB, BLCAP, ZBTB47, PKIA,* and *IGF2*. Among them, *MITF* is an inhibitor of hypoxia-inducible factor, which can promote angiogenesis. Therefore, the loss of *MITF* function may result in the angiogenesis of malignant tumours^39^. *THRB* is a nuclear hormone receptor for triiodothyronine and thus, could promote the biological activities of thyroid hormone (T3), such as the differentiation, growth, development, and maintenance of metabolic homeostasis. *THRB* reduces the abundance of *VEGF* in tumour cells; thus, its abnormal downregulation may lead to enhanced tumour angiogenesis^40^. At the posttranscriptional level, the upregulation of hsa-miR-425 inhibits the translation of *THRB* mRNA^41,42^, which is consistent with our results. *BLCAP* inhibits the cell cycle, induces apoptosis as an apoptotic inducer, and acts as a tumour suppressor gene^43,44^. *ZBTB47* encodes a transcription factor with a zinc finger domain, which plays a role in inhibiting transcription^45^. Its homologous transcription factor is the tumour suppressor gene of breast cancer; thus, *ZBTB47* may be a tumour suppressor^46^. *IGF2*^47–51^ and *PKIA*^52^ are highly expressed in tumour cells and promote tumour progression. However, our results suggest that these two genes are downregulated in osteosarcoma tissues and can be inhibited by the upregulation of hsa-miR-9-3p and hsa-miR-155-5p. These results suggest that *IGF2* and *PKIA* may not be involved in the progression of osteosarcoma. Moreover, aberrantly downregulated lncRNAs regulate key genes involved in angiogenesis, apoptosis inhibition, and transcription.

In this study, RNA sequencing of osteosarcoma tissues and matched paratumour tissues was performed to systematically explore aberrantly expressed lncRNAs in osteosarcoma tissues and their role in regulating key genes via the ceRNA network. Some bias exists because of the small sample size. In addition, further clinical trials may be needed to verify the results.

## Conclusion

Aberrantly expressed lncRNAs in osteosarcoma tissues regulate genes involved in cellular proliferation, differentiation, angiogenesis, and the cell cycle via the ceRNA network.

## Supplementary Information

Supplementary Figure 1.
