# Supplementary material for: The regulatory mechanism of LncRNA-mediated ceRNA network in osteosarcoma
Source: Sci Rep. 2022 May 24;12:8756. doi: 10.1038/s41598-022-11371-w (PMC9130241; doi:10.1038/s41598-022-11371-w)
Supplement: Supplementary file 1 — Supplementary Figure 1. [file 41598_2022_11371_MOESM1_ESM.docx]

Table S1 The 20 lncRNAs with the largest expression differences

| lncRNA | logFC | logCPM | PValue | FDR |
| --- | --- | --- | --- | --- |
| LINC01419 | 11.20134 | 11.05999 | 0.000247 | 0.003182 |
| LINC02475 | 10.63579 | 10.50224 | 5.60E-14 | 1.72E-11 |
| LINC01583 | 8.9838 | 8.912657 | 2.84E-06 | 9.39E-05 |
| SATB2-AS1 | 8.80793 | 8.741966 | 4.94E-05 | 0.000818 |
| LINC01549 | 8.563085 | 8.513549 | 3.65E-06 | 0.000109 |
| TTN-AS1 | 8.215157 | 8.197998 | 4.76E-09 | 3.65E-07 |
| AL645608.2 | 8.188799 | 8.170481 | 7.43E-07 | 2.80E-05 |
| LINC01614 | 8.060496 | 9.788491 | 2.08E-11 | 2.98E-09 |
| AL157702.2 | 8.039111 | 8.038914 | 4.17E-08 | 2.19E-06 |
| AC004988.1 | 8.021124 | 8.029354 | 1.32E-05 | 0.000294 |
| AC079467.1 | -12.5994 | 12.44438 | 6.34E-23 | 6.82E-20 |
| MIR133A1HG | -12.4427 | 13.92861 | 9.03E-28 | 1.94E-24 |
| MIR1-1HG | -10.6069 | 11.56067 | 4.36E-16 | 2.34E-13 |
| AC015878.1 | -9.91707 | 9.799813 | 2.39E-13 | 5.70E-11 |
| AC068506.1 | -9.47006 | 9.369053 | 4.86E-13 | 9.49E-11 |
| LINC01405 | -9.42916 | 10.31227 | 6.24E-09 | 4.62E-07 |
| AL451062.1 | -9.40565 | 9.307296 | 1.11E-13 | 2.98E-11 |
| HSD52 | -9.28552 | 9.192424 | 5.23E-12 | 8.65E-10 |
| AC011239.1 | -9.26059 | 10.72637 | 4.62E-13 | 9.49E-11 |
| KIAA1671-AS1 | -8.74055 | 10.21971 | 2.17E-14 | 7.79E-12 |

Table S2 The 20 miRNAs with the largest expression differences

| miRNA | logFC | logCPM | PValue | FDR |
| --- | --- | --- | --- | --- |
| hsa-miR-31-3p | 9.594772 | 2.387346 | 0.000223 | 0.005377 |
| hsa-miR-592 | 9.544012 | 2.335825 | 5.59E-05 | 0.002024 |
| hsa-miR-767-3p | 9.051203 | 1.870478 | 7.44E-06 | 0.000462 |
| hsa-miR-105-3p | 8.882965 | 1.711551 | 1.78E-06 | 0.000155 |
| hsa-miR-181b-3p | 8.739779 | 1.573519 | 3.06E-05 | 0.001423 |
| hsa-miR-520e | 8.540185 | 1.401109 | 0.001791 | 0.019303 |
| hsa-miR-1269b | 8.499618 | 7.577274 | 8.70E-06 | 0.000493 |
| hsa-miR-138-5p | 8.295049 | 5.659418 | 0.000111 | 0.003072 |
| hsa-miR-105-5p | 8.194404 | 4.724987 | 2.36E-08 | 2.56E-06 |
| hsa-miR-216b-5p | 8.189633 | 3.786986 | 0.000562 | 0.009269 |
| hsa-miR-133a-3p | -10.73 | 15.55795 | 1.93E-16 | 2.16E-13 |
| hsa-miR-133a-5p | -10.4162 | 6.81363 | 3.81E-11 | 9.95E-09 |
| hsa-miR-206 | -10.3872 | 16.2581 | 3.16E-12 | 1.37E-09 |
| hsa-miR-133b | -9.86116 | 11.36755 | 3.32E-16 | 2.16E-13 |
| hsa-miR-1-3p | -9.45035 | 15.63407 | 2.44E-11 | 7.95E-09 |
| hsa-miR-499b-3p | -9.05284 | 10.70175 | 1.78E-09 | 2.48E-07 |
| hsa-miR-499a-5p | -9.04572 | 10.71222 | 1.90E-09 | 2.48E-07 |
| hsa-miR-128-2-5p | -9.0343 | 1.835869 | 8.00E-06 | 0.000474 |
| hsa-miR-208b-3p | -8.91467 | 1.714608 | 0.000334 | 0.006753 |
| hsa-miR-885-3p | -8.11682 | 0.979582 | 3.45E-06 | 0.000237 |

Table S3 The 20 mRNAs with the largest expression differences

| mRNA | logFC | logCPM | PValue | FDR |
| --- | --- | --- | --- | --- |
| IFITM5 | 14.35229 | 7.561673 | 1.48E-10 | 5.44E-09 |
| PANX3 | 12.79836 | 7.82507 | 5.34E-12 | 2.48E-10 |
| IBSP | 11.86124 | 9.934298 | 8.95E-12 | 3.98E-10 |
| AMBN | 11.81128 | 7.200701 | 1.16E-07 | 2.27E-06 |
| MAGEC2 | 11.42705 | 4.650217 | 6.11E-06 | 7.46E-05 |
| SSX1 | 11.24453 | 4.469862 | 1.18E-07 | 2.31E-06 |
| DMP1 | 11.1683 | 5.843946 | 2.92E-07 | 5.17E-06 |
| SOST | 11.02 | 7.052596 | 1.35E-07 | 2.59E-06 |
| EPYC | 10.84813 | 4.079108 | 3.91E-09 | 1.06E-07 |
| MMP7 | 10.59979 | 3.835182 | 3.12E-07 | 5.48E-06 |
| MYH7 | -16.7415 | 14.65585 | 2.12E-20 | 2.58E-18 |
| MYH2 | -16.0602 | 10.73007 | 3.04E-38 | 4.81E-35 |
| MYBPC1 | -15.5262 | 10.36916 | 2.82E-40 | 6.37E-37 |
| NRAP | -15.0091 | 10.33261 | 1.73E-33 | 1.09E-30 |
| TNNC2 | -14.9977 | 10.29801 | 9.30E-43 | 4.90E-39 |
| XIRP2 | -14.4986 | 10.10527 | 1.59E-34 | 1.67E-31 |
| MYH1 | -14.4975 | 13.26855 | 1.35E-53 | 2.14E-49 |
| AMPD1 | -14.2874 | 7.496955 | 6.35E-35 | 7.16E-32 |
| MYL2 | -14.2619 | 11.34876 | 4.28E-16 | 3.40E-14 |
| PLA2G2A | -14.0994 | 7.309162 | 7.94E-15 | 5.46E-13 |
